# Indian research on sleep disorders

**DOI:** 10.4103/0019-5545.69242

**Published:** 2010-01

**Authors:** Nilesh Shah, Abha Bang, Aparna Bhagat

**Affiliations:** Department of Psychiatry, L. T. M. Medical College and General Hospital, Sion, Mumbai - 400 022, India

**Keywords:** Sleep Disorders, Indian literature on sleep disorders

## Abstract

Literature on sleep disorders from our country, India, can mainly be found in the Indian journal of Sleep medicine, Indian Journal of psychiatry, The Annals of Indian Academy of Neurology and certain other journals and books. The article highlights the contribution of various Indian doctors in the field of sleep disorders, which includes review articles, prevalence studies, studies on etiology and treatment options, case reports and a couple of case control studies. Also included are studies on various sleep related syndromes as well as studies about awareness and knowledge of sleep disorders amongst the medical fraternity. This is a humble attempt to compile the rich data available in the country on sleep disorders in order to aid further research in the field.

Sleep disorder literature from our country, India, is largely published in the Indian Journal of Sleep Medicine, Indian Journal of Psychiatry, The Annals of Indian Academy of Neurology and certain other journals and books. It is in the form of review articles, studies on etiology and treatment options, case reports and a couple of case control studies. This article brings together the rich data on sleep disorders available in the country in order to aid further research in the field.

The international classification of sleep disorders-2 (2005) (ICSD-2) classifies the sleep disorders in six major categories:[1]

- Insomnia
- Sleep related breathing disorders
- Hypersomnias
- Circadian rhythm sleep disorder
- Parasomnia
- Sleep related movement disorders

We have tried to classify the available data according to these broad categories along with another category for those articles which could not fit into any of the above sections.

## INSOMNIA

Insomnia refers to the difficulty in initiation, maintenance, duration or quality of sleep. People may experience poor concentration, lower productivity and poorer work quality as a result of insomnia.

Studies highlighting novel pharmacological agents for treating this disorder included the one on Ramelteon a novel MT1 and MT2 melatonin receptor selective agonist recently approved for the treatment of insomnia characterized by difficulty in sleep onset. According to a study by Devi V and Shankar PK, so far there is no evidence of cognitive impairment, rebound insomnia, withdrawal effects or abuse potential with the use of Ramelteon.[2]

In another multi-centric comparative clinical trial done on 100 patients, Phadke S and Shetty Jyoti, found that Eszopiclone was effective and comparable to other treatment in improving the sleep parameters in patients suffering from insomnia with a better safety profile than Zopiclone, Zolpidem and Nitrazepam.[3] Also, an interesting case report by Bhat T, Pallikaleth SJ and Shah N showed the successful use of Zolpidem for treatment of an 18-month- old child suffering from primary insomnia.[4]

In an attempt to generate awareness and spread knowledge about sleep disorders encountered with various other general medical conditions in routine practice, a series of books on insomnia in various clinical setting and its treatment have been published. These books were mainly intended for distribution amongst general physicians, orthopedicians, neurologists, psychiatrists, chest physicians and other specialties. These books include the management of insomnia in pain, chronic obstructive pulmonary disease, diabetes mellitus, and cardiovascular diseases.[5–8]

## SLEEP RELATED BREATHING DISORDERS

Sleep related breathing disorders include several potentially serious conditions that include primary snoring, Upper airway resistance syndrome (UARS), obstructive sleep apnea-hypopnea syndrome (OSAHS), central sleep apnea, asthma and chronic obstructive pulmonary disease.

An effort to identify potential perception for genetic and biochemical basis of risk factors of OSAS and related co- morbidities was made by Kant surya *et al*. in their study published in 2008.[9] Kanwar M.S, in a comparative study on upper airway resistance syndrome (UARS) and obstructive sleep apnoea (OSA), discovered that UARS is not a distinct syndrome. It is merely a part of the disease spectrum of OSAHS. There exists a group that has characteristics of both OSA and UARS. The spectrum of disease is Primary snorers (PS) à UARS à combined (OSA+UARS) and combined (OSA+OSAHS). UARS patients were mostly non-obese with active life style as compared to OSA patients. Also they displayed poor sleep efficiency with more prolonged awakenings compared to OSA patients.[10]

In an unusual study, Ambar Chakravarthy describes his personal experience of systemic effects of late night sleep deprivation and non-restorative sleep - A common experience amongst doctors. Results of some simple self‑experimentations have been mentioned to highlight the possible pathogenetic mechanisms.[11]

Due to the paucity of data on Indian children with sleep disordered breathing (SDB), a study to evaluate the common clinical presentation, etiological factors, radiological and polysomnographic features in these children presenting to a tertiary care hospital was carried out by Bhool Shikha *et al*.[12]

In an attempt to find an effective and easy screening method for OSA, Nag Saikat *et al*. used the measurement of cephalic shadow to predict obstructive sleep apnoea, but found that there was no statistically significantly co- relationship with apnea-hypopnea index (AHI) and any of the cephalometric measurements.[13] In a case control study on OSA, Pradeep Kumar VG *et al*. found that patients with OSA had significantly higher BMI and ESS score, and were more likely to have hypertension and road traffic accidents.[14] The frequency, clinical and polysomnographic profile of sleep apnea syndromes seen in neurologic practice was studied by Kaul S, Meena AK and Murthy JM. According to their experience, obstructive apnea is the commonest sleep apnea in India and occurs predominantly in middle aged males.[15]

Zarir F. Udwadia *et al*. in a first-of-its-kind two-phase cross-sectional prevalence study found that the prevalence of SDB was 19.5%, and that of OSAHS was 7.5% in healthy urban Indian males between 35-65 years of age. The findings of this study and the high prevalence rates in middle-aged urban Indian men might have important public health implications in a developing country with limited health resources.[16]

Suri J.C. and Sen M.K. studied the pulmonary functions in obstructive sleep apnoea hypopnoea syndrome in 89 patients attending the sleep centre of a tertiary care hospital.[17] Aggarwal Manju *et al*. in a study on maternal and fetal outcomes of sleep disordered breathing (SDB) in pregnancy concluded that SDB has widespread systemic effects that include adverse impact on maternal and fetal outcomes of pregnancy.[18]

Bhattacharya D. *et al*. presented a child with Pierre Robin Syndrome (PRS) - A rare genetic disorder characterized by micrognathia, cleft palate and glossoptosis; in whom sleep study performed showed presence of significant sleep disordered breathing. It also showed the effects of SDBs on physical and mental development.[19] Gangurde Aniket, Gothi D, Joshi JM in 2006 reported a case of a 13-year-old girl with postaxial acrofacial dysostosis (POADS) also called Miller Syndrome with sleep disordered breathing due to bilateral temporomandibular joint (TMJ) ankylosis and micrognathia/retrognathia, which was effectively treated with surgical correction.[20]

Jayan B *et al*. observed short term therapeutic efficacy of oral appliances both clinically and by PSG studies. According to them, Oral appliances therapy for OSA is non invasive, cost effective and beneficial to affected patients if desired efficacy is achieved. It greatly improves quality of life and cardio pulmonary health.[21] In another pilot study to evaluate the therapeutic efficacy of Thornton adjustable positioner (TAP), a titratable MAD (mandibular advancement appliances) in severe adult OSA cases, Jayan B *et al*. concluded that factoring predictable cephalometric measurements, BMId ≤30 kg/m^2^, and mandibular protrusion of 70%; severe OSA can be effectively managed with TAP.[22]

Suri J.C., Sen M.K. and Ojha U.C. studied the acceptance and compliance issues of Nasal cap amongst Indian patients of obstructive sleep apnoea. Several factors responsible for non acceptance and poor compliance were identified, which included causes related to social, economic, cultural and geographical parameters peculiar to our country.[23] Another recently developed technique of Distraction osteogenesis has been found to be valuable and gives us the ability to both prevent and correct the development of sleep disordered breathing as found by Chowdhury S.K. Roy *et al*. in a study done in 2007.[24]

## HYPERSOMNIA

Excessive daytime sleepiness, defined as sleepiness that interferes with daytime activities, productivity or enjoyment is usually abnormal and may reflect insufficient sleep, disrupted sleep or a primary sleep disorder such as narcolepsy.

Bhatia M and Arif MA reported probably the first documented case of narcolepsy, from India, diagnosed on the basis of clinical history and sleep studies in 2009.[25] Anjan Boral and Nilesh Shah have reported a case of Klein Levine Syndrome in 1994.[26] D.N Mendhekar *et al*. reported two cases of Kleine Levin Syndrome in 2001.[27] John DJ *et al*. presented a case in 2007, that highlights the clinical presentation, diagnostic criteria and treatment modalities of primary hypersomnia condition.[28] Bihari S. and Ramakrishnan N reported the case of a boy with episodic hyper somnolence.[29] Chittaranjan Andrade studied the response to fluoxetine and methylphenidate in primary hypersomnia.[30]

## CIRCADIAN RHYTHM SLEEP DISORDERS

Circadian rhythm sleep disorders include the sleep disorders in which sleep-wake cycle is disturbed as it happens in workers having shift duties and in travelers who get a jetlag.

The pattern of sleep, prevalence of anxiety and depression and the overall impact of the nature of their employment on their lifestyle were studied in a segment of BPO workers employed in the call centers around New Delhi by Suri J.C. *et al*. in 2007. It was found that Circadian rhythm sleep disorders (CRSD) are not infrequently seen amongst shift workers who, in turn, comprise a large segment of the population employed in the BPO industry.[31]

## PARASOMNIA

Parasomnia is an undesirable nondeliberate motor or subjective phenomenon that takes place during transition from wakefulness to sleep or during arousal from sleep.

Probably the first ever study in India on sleep paralysis was conducted by Jaswant Singh Neki in the year 1961.[32] Bharadwaj R and Kumar S presented two cases of somnambulism that highlight the importance of the diagnosis and treatment of this condition.[33] Rajesh, *et al*. with the help of a case-report of a 30 year old male patient had highlighted the fact that the onset of sleep walking in adult life which is most unusual suggests the presence of secondary causes rather than a primary sleep disorder; as in this case where the episodes of sleep walking were possibly due to nocturnal temporal lobe epilepsy.[34] Sawant NS *et al*. reported the case of a patient with isolated sleep paralysis who progressed from mild to severe sleep paralysis over eight years. He also restarted drinking alcohol to be able to fall asleep and allay his anxiety symptoms. The patient was taught relaxation techniques and he showed complete remission of the symptoms of SP on follow-up after eight months.[35]

## SLEEP RELATED MOVEMENT DISORDERS

A variety of involuntary movements occur during the sleep and these are covered under this category of sleep related movement disorders.

Periodic limb movement disorder (PLMD) is one of the commonest neurological disorders and causes significant disability, if left untreated. However, it is rarely diagnosed in clinical practice, probably due to lack of awareness and/or lack of necessary diagnostic facilities. Restless leg syndrome (RLS), aging, pregnancy, uremia, iron deficiency, polyneuropathy are some of the common causes of secondary PLMD. Clinical presentation, polysomnographic findings and management of six patients of PLMD have been discussed by Dhanuka AK and Singh G.[36] Restless legs syndrome (RLS) is a disorder of motor activity with a circadian pattern, occurring frequently in patients with Parkinson’s disease (PD). Krishnan PR, Bhatia M and Behari M attempted to estimate the prevalence of RLS in Indian PD patients and found that RLS is more common among patients with PD than controls.[37] Restless-legs syndrome and periodic leg movements in sleep are sleep-related limb movement disorders that often disrupt nocturnal sleep and cause excessive daytime sleepiness. The article by Samavedam A. and Krishna reviews the recent literature regarding epidemiology, etiology, pathogenesis, clinical features, differential diagnosis and management.[38]

## OTHER RELATED ARTICLES

Indian literature also has a variety of case reports and studies which are related to sleep and sleep disorders.

Bhargav S.C. and Sethi S in their study evaluating 32 children suffering from Attention Deficit Hyperkinetic Disorder (ADHD) and their 20 healthy siblings inferred that sleep-related problems may have a significant bearing on the course and management of ADHD and that a careful evaluation of sleep history is recommended in these children.[39]

A study by Suri J.C., *et al*. was done to analyze the epidemiology of sleep disorders in the elderly population. This exhaustive study included 1240 grand-parents of school-going children in Delhi.[40] Also, Suri J.C., *et al*. using the Chervin and the Stanford Sleep Clinic questionnaire had conducted a study on a sample of adult population of Delhi which reflected that the impact of sleep disorders on the morbidity profile on this strata of society, the phenomenal burden of undiagnosed sleep disorders and its impact on social, mental, physical and economic health of the society.[41] Another questionnaire based survey by Suri J.C., *et al*. was performed to determine the prevalence of sleep related disorders in Indian school-going children residing in Delhi. It was of great concern that the findings suggested that no effort was made on the part of parents to seek medical help in significantly large number of children in whom sleep disorders were present, indicating a total lack of awareness amongst the general population about the larger implications of sleep disorders in children.[42] Narendhran R, *et al*. designed a study to assess the psychometric properties of a parent-rated measure of sleep habits i.e. Children Sleep Habits Questionnaire (CSHQ) in Indian school going children, concluding that CSHQ is a reliable and internally consistent scale, and it is useful optional tool for assessing sleep problems in Indian school children.[43]

A study was conducted by Ghoshal A.G., *et al*. in the Department of Respiratory Medicine, Nil Ratan Sircar Medical College, Kolkata over a period of two years on 120 uncomplicated stable adult asthmatics to assess the incidence of Excessive Daytime Sleepiness (EDS) in patients with asthma, and to find out its correlation with the severity and level of control of asthma. A definite correlation was found between EDS with the severity of asthma and the level of control but not with the mode of diagnosis of asthma.[44]

One study by Krishna Pushpa, Shwetha S on 67 medical students aimed to analyze the quality of sleep in medical students using the Pittsburgh Sleep Quality Index and to relate sleep with blood pressure (BP), body mass index (BMI) and academic performance. This study shows the high prevalence of poor sleep quality and underlined the close relationship of sleep with BP, BMI and academic performance among medical students.[45] Meshram Sushant H *et al*. after having conducted a questionnaire-based study to assess the behavior, attitude and knowledge of sleep medicine among resident doctors had concluded that there was an intense need for including sleep medicine in their curriculum.[46]

Based on a comparative hospital study based on electroencephalographic and radiological profiles of the subjects, by Goel D, *et al*., it was found that it is likely that dominant side and frontal lobe involvement in symptomatic epilepsies is associated with higher number of seizures during sleep and that techniques like sleep EEG, sleep deprived EEG and video-EEG telemetry are supposed to improve outcome in the diagnosis of patients with sleep seizures. Study on clinical, electroencephalographic and radiological profile.[47] by Bhatia M, *et al*. aimed to translate the Epworth Sleepiness Scale into Hindi and validate it for use in the Hindi speaking population in India. With the help of a study conducted by them the Hindi version was found to be valid and reliable for use in the evaluation of sleepiness in Hindi speaking population of our country.[48]

S. R. Iyer and R. R. Iyer in their article, ‘Sleep and Ageing - Interactions and Consequences’ have described sleep patterns changing subjectively and polysomnographically with ageing.[49] In yet another article they describe the existence of a close and interesting relationship amongst sleep, OSA, obesity, insulin resistance and metabolic syndrome.[50]

A case report on electrical Status Epilepticus of Sleep was authored by Nilesh Shah and Kedar Kale.[51] Mahesh Bhirud and Nilesh Shah in 2004 presented a case-reports of Clozapine induced urinary incontinence during sleep which were treated with imipramine.[52] The handbook of sleep, authored by Dr. Nilesh Shah, *et al*. provides a comprehensive information on sleep and the various therapies recommended for sleep and is a handy reference for clinicians, helping them tackle the challenges of the increasing incidence of insomnia in the general population.[53]
